# Good Glycemic Control Is Associated with Better Survival in Diabetic Patients on Peritoneal Dialysis: A Prospective Observational Study

**DOI:** 10.1371/journal.pone.0030072

**Published:** 2012-01-23

**Authors:** Dong Eun Yoo, Jung Tak Park, Hyung Jung Oh, Seung Jun Kim, Mi Jung Lee, Dong Ho Shin, Seung Hyeok Han, Tae-Hyun Yoo, Kyu Hun Choi, Shin-Wook Kang

**Affiliations:** Department of Internal Medicine, College of Medicine, Brain Korea 21 for Medical Science, Severance Biomedical Science Institute, Yonsei University, Seoul, Korea; National Cancer Institute, United States of America

## Abstract

**Background:**

The effect of glycemic control after starting peritoneal dialysis (PD) on the survival of diabetic PD patients has largely been unexplored, especially in Asian population.

**Methods:**

We conducted a prospective observational study, in which 140 incident PD patients with diabetes were recruited. Patients were divided into tertiles according to the means of quarterly HbA1C levels measured during the first year after starting PD. We examined the association between HbA1C and all-cause mortality using Cox proportional hazards models.

**Results:**

The mean age was 58.7 years, 59.3% were male, and the mean follow-up duration was 3.5 years (range 0.4–9.5 years). The mean HbA1C levels were 6.3%, 7.1%, and 8.5% in the 1^st^, 2^nd^, and 3^rd^ tertiles, respectively. Compared to the 1^st^ tertile, the all-cause mortality rates were higher in the 2^nd^ [hazard ratio (HR), 4.16; 95% confidence interval (CI), 0.91–18.94; p = 0.065] and significantly higher in the 3^rd^ (HR, 13.16; 95% CI, 2.67–64.92; p = 0.002) tertiles (p for trend = 0.005), after adjusting for confounding factors. Cardiovascular mortality, however, did not differ significantly among the tertiles (p for trend = 0.682). In contrast, non-cardiovascular deaths, most of which were caused by infection, were more frequent in the 2^nd^ (HR, 7.67; 95% CI, 0.68–86.37; p = 0.099) and the 3^rd^ (HR, 51.24; 95% CI, 3.85–681.35; p = 0.003) tertiles than the 1^st^ tertile (p for trend = 0.007).

**Conclusions:**

Poor glycemic control is associated with high mortality rates in diabetic PD patients, suggesting that better glycemic control may improve the outcomes of these patients.

## Introduction

Diabetes mellitus (DM) is the leading cause of end-stage renal disease (ESRD) worldwide, accounting for more than 40% of incident dialysis patients in the United States [1]. To delay diabetic nephropathy from progressing and to improve outcomes for DM patients, a multidisciplinary approach is currently recommended, including glycemic control [2].

Accumulating evidences have shown that tight glycemic control prevents the development and progression of diabetic complications in both type 1 and type 2 DM patients [3]–[5]. In addition, high blood glucose concentrations were found to be associated with increased incidence of cardiovascular disease in diabetic patients [6]. Moreover, HbA1C levels were revealed as an independent risk factor for coronary heart disease in diabetic patients [7]. Since cardiovascular diseases are the most common cause of death in DM patients, it has been surmised that strict glucose control may be favorable to the outcome in these patients. However, recent several randomized controlled trials have failed to demonstrate any beneficial effects of strict glycemic control on the cardiovascular morbidity and mortality in type 2 DM patients without advanced renal failure [8]–[10].

While many previous studies have excluded diabetic patients with advanced renal failure, only a few investigations have explored the impact of glycemic control on the prognosis of DM patients on dialysis, with inconsistent results [11]–[14]. An American report using a database from a large dialysis organization showed a significant correlation between the levels of HbA1C and prognosis in diabetic patients on hemodialysis (HD) [13], while another recent Canadian study found that higher blood glucose and HbA1C levels were not associated with mortality in maintenance HD patients with DM [14]. Different from HD, peritoneal dialysis (PD) results in a large amount of glucose load that is continuously absorbed from the dialysate. Therefore, glycemic control may be more difficult, and the impact of strict glycemic control on the clinical outcomes may be more obvious in diabetic PD patients, but definite evidence is furthermore lacking in these patients. To date, only one study has investigated the relationship between glycemic control after starting PD and the clinical outcomes in type 2 diabetic PD patients, in which only a few Asians were included [15]. Although there has been a study conducted in Asian population to show the association between glycemic control and patient outcomes, glycemic control before starting dialysis was used as an indicator of glycemic control [16]. In this study, we tried to determine whether glycemic control after starting PD was associated with all-cause and cardiovascular mortality in Asian diabetic PD patients.

## Methods

### Ethics statement

This study was approved by the Institutional Review Board for human research at Yonsei University College of Medicine, and all participants provided their written informed consent prior to study entry.

### Study setting and participants

For this prospective observational study, we recruited 145 incident continuous ambulatory PD patients with DM from a single Korean dialysis center, and followed them at Yonsei University Health System in Seoul, Korea. Enrollment of patients was conducted from Jan 2001 until December 2008. The diagnosis of DM at the initiation of PD was based on the diagnostic criteria of the American Diabetes Association [17]. We excluded patients who were younger than 20 years old (n = 1), had a history of malignancy (n = 1), a history of receiving a kidney transplant (n = 1), or a history of HD for more than three months (n = 1). Patients who failed to maintain PD for more than three months were also excluded (n = 1).

### Data Collection

To assess glycemic control, monthly preprandial blood glucose and quarterly HbA1C levels were collected during the first year after starting PD. However, to exclude the possibility of undue hyperglycemia, the HbA1C levels were omitted from mean HbA1C levels when measured during acute illness or when taking medications such as glucocorticoid that can affect blood glucose concentrations. Blood glucose concentrations were determined by the hexokinase-UV method and HbA1C levels were measured by high-performance liquid chromatography. The mean preprandial blood glucose and HbA1C values were used for this analysis.

The following demographic and clinical data were collected for each patient at the beginning of PD: age, gender, height, weight, body mass index (BMI), primary renal disease, duration of DM, smoking status, and comorbid conditions including hypertension, chronic lung disease, chronic liver disease, cardiovascular disease (CVD), and other serious medical illnesses. CVD included coronary artery disease, peripheral vascular disease, and cerebrovascular disease. The Charlson comorbidity index (CCI) score was used to quantify comorbid conditions [18]. Information on blood pressure and antihypertensive medications was collected at 3 months after beginning PD, when the patients' volume status had stabilized. The management of hyperglycemia was categorized into 4 groups; no medication, oral hypoglycemic agents alone, insulin alone, and combined treatment (oral hypoglycemic agents and insulin). The following laboratory data were also measured from blood samples taken 3 months after beginning PD: hemoglobin, white blood cell count, blood urea nitrogen, creatinine, albumin, calcium, phosphorus, intact parathyroid hormone (iPTH), total cholesterol, uric acid, bicarbonate, and high sensitivity c-reactive protein (hsCRP). Residual GFR was calculated as the average of urea and creatinine clearance from a 24-hour urine collection. Kt/V_urea_ was determined from the total urea nitrogen loss in the spent dialysate using the Watson equation [19], and normalized protein catabolic rate (nPCR) [20] was assessed for nutritional status.

### Outcomes

Patients were classified into tertile groups, based on their average HbA1Cs during the first year after beginning PD, and prospectively followed from enrollment until death, transfer to an alternative dialysis method, or Dec 2010. Patients who transferred to HD or transplantation were censored for the patient survival analysis. The primary and secondary outcomes for all analyses were all-cause and cardiovascular mortality, respectively.

### Statistical analysis

Statistical analysis was performed using SPSS version 13.0 (SPSS, Inc., Chicago, Illinois, USA). Data were basically expressed as mean ± standard deviation (SD) or percentages. Due to the log-normal distributions of hsCRP and iPTH, natural log values were used for analyses. Geometric means for all log-normally distributed continuous variables were calculated and reported with geometric SD. Results were analyzed using ANOVA or chi-square tests for comparisons. Significant differences detected by ANOVA were further confirmed by the Student's t-tests with the Bonferroni corrections. The relationships between HbA1C and preprandial blood glucose or log-transformed hsCRP (log hsCRP) levels were determined by Pearson's correlation analysis. Cox proportional hazards analysis was performed on variables revealed to be significant by univariate analysis to define the effect of HbA1C levels on mortality. A case-mix model was performed after adjusting for age, gender, year of PD start, CCI score. In the fully-adjusted model, mean arterial pressure (MAP), serum creatinine, albumin, and log hsCRP levels were further adjusted in addition to all variables used in the case-mix model. P-values less than 0.05 were considered statistically significant.

## Results

### Baseline characteristics and laboratory findings of patients

**Figure 1:**
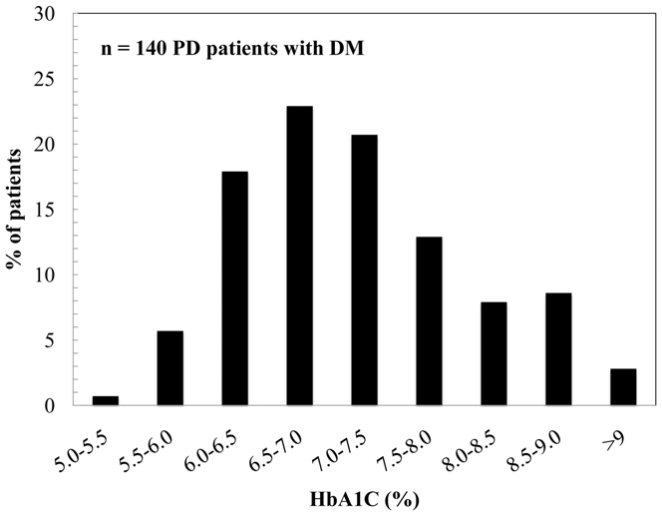
The frequency distribution of HbA1C values for all study patients.

Of the 810 patients who began PD between January 2001 and December 2008, 145 patients had DM. After excluding 5 patients, a total of 140 patients were finally recruited in this study. The baseline characteristics of the study patients are shown in Table 1. The mean age was 58.7 years, 59.3% were male, and the mean follow-up duration was 3.5 years (range 0.4–9.5 years). The primary renal diseases were diabetic nephropathy (85.0%), chronic glomerulonephritis (7.1%), and hypertensive nephrosclerosis (4.3%) in order. Hypertension and CVD were accompanied in 139 (99.3%) and 44 (31.4%) patients, respectively. The mean systolic and diastolic blood pressures were 133.9±19.4 and 77.5±11.5 mmHg, respectively, and 75.7% of patients were taking RAS blockades. The frequency distribution of HbA1C values for all study patients is shown in Figure 1, and 47.1% of patients were within the recommended target HbA1C (less than 7%). Hypoglycemia occurred at the frequency of 1.1 events per 100 patient-year.

**Table 1:** Comparision of demographic, clinical, and laboratory characteristics in each tertile.

		I (5.15–6.7)	II (6.8–7.5)	III (7.6–13.25)	P
	n = 140	n = 46	n = 47	n = 47	
Age, years (SD)	58.7±10.6	57.2±11.5	59.2±9.2	59.6±11.0	0.493
Male gender	83 (59.3%)	33 (71.7%)	30 (63.8%)	20 (42.6%)	0.012
Follow-up duration, years	3.5±2.0	3.6±1.9	3.9±2.0	3.0±1.9	0.095
Diabetes as the cause of ESRD	119 (85.0%)	37 (80.4%)	40 (85.1%)	42 (89.4%)	0.105
CVD	44 (31.4%)	18 (39.1%)	10 (21.3%)	16 (34.0%)	0.160
CCI score	5.8±1.4	5.6±1.4	5.8±1.2	6.0±1.7	0.352
Year of starting PD					0.306
2001∼2004	45 (32.1%)	12 (26.1%)	14 (29.8%)	19 (40.4%)	
2005∼2008	95 (67.9%)	34 (73.9%)	33 (70.2%)	28 (59.6%)	
BMI (kg/m^2^)	23.2±2.7	23.4±3.0	23.4±2.4	22.8±2.8	0.489
Systolic BP (mmHg)	133.9±19.4	134.1±19.2	135.2±21.2	132.4±17.9	0.796
Diastolic BP (mmHg)	77.5±11.5	77.8±11.0	78.2±11.0	76.6±12.6	0.778
Methods of glycemic control					0.135
Insulin	55 (39.3%)	17 (37.0%)	18 (38.3%)	20 (42.6%)	
Oral hypoglycemic agent	59 (42.1%)	24 (52.2%)	20 (42.6%)	15 (31.9%)	
Combined	19 (13.6%)	3 (6.5%)	5 (10.6%)	11 (23.4%)	
No control	7 (5.0%)	2 (4.3%)	4 (8.5%)	1 (2.1%)	
Hypoglycemic event*	1.1	0.9	1.1	1.2	0.250
Hemoglobin (g/dL)	11.0±1.7	11.0±1.8	11.1±1.8	10.9±1.5	0.842
HbA1C (%)	7.3±1.1	6.3±0.3	7.1±0.3	8.5±1.1	<0.001
Preprandial glucose (mg/dL)	145.3±50.3	104.9±22.6	136.2±16.6	194.0±52.2	<0.001
Creatinine (mg/dL)	6.6±2.4	6.9±2.6	6.9±2.7	6.0±1.9	0.100
Albumin (g/dL)	3.3±0.5	3.4±0.4	3.4±0.4	3.1±0.5^(I,II)^	0.003
Total cholesterol (mg/dL)	184.1±44.6	178.7±45.6	180.2±38.1	193.3±49.0	0.220
Bicarbonate (mmol/L)	27.7±3.1	27.7±3.0	27.6±3.2	28.0±3.3	0.821
Calcium (mg/dL)	8.9±0.9	8.9±1.0	9.1±0.8	8.9±0.9	0.411
Phosphorus (mg/dL)	4.2±1.0	4.4±1.0	4.2±0.9	4.0±0.9	0.125
iPTH (pg/mL)#	74.9±3.5	98.2±4.1	70.0±3.5	59.3±2.9	0.245
hsCRP (mg/L)#	1.57±5.38	1.60±5.37	1.31±5.02	1.83±5.85	0.654
Total Kt/V*_urea_*	2.48±0.62	2.37±0.61	2.54±0.68	2.55±0.58	0.450
RRF (ml/min/1.73 m^2^)	4.62±3.20	4.59±2.49	4.50±3.88	4.76±3.38	0.953
nPCR (g/kg/day)	0.97±0.21	0.95±0.21	1.04±0.21	0.94±0.20	0.120

Data are presented as mean ± SD or n (%).

# expressed as geometric mean ± geometric SD. ESRD, end-stage renal disease; CVD, cardiovascular disease; CCI, Charlson comorbidity index; PD, peritoneal dialysis; BMI, body mass index; BP, blood pressure; iPTH, intact parathyroid hormone; hsCRP, high-sensitivity C-reacitve protein; RRF, residual renal function; nPCR, normalized protein catabolic rate.

*per 100-patient year.

During the follow-up, 23 (16.4%) patients died, 28 (20.0%) were transferred to HD, and 7 (5.0%) received a kidney transplant. Cardiovascular disease (39.1%) and infection (39.1%) were the most common causes of death. Among death due to infection, PD-related infection such as PD peritonitis accounted for only 22.2% of all infection-related death, while non-PD-related causes, including pneumonia, wound infection, and necrotizing colitis, contributed to the majority of infection-related death (77.8%).

### Correlation between preprandial blood glucose and HbA1C

**Figure 2:**
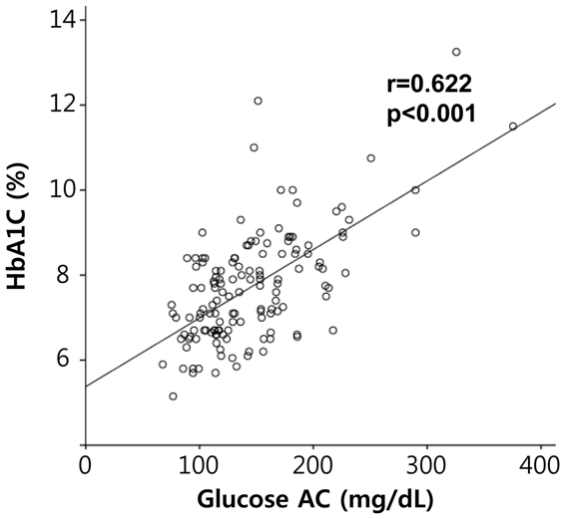
Bivariate correlation analysis between HbA1C and preprandial glucose (Glucose AC).

Pearson's correlation analysis revealed a significant correlation between preprandial blood glucose and HbA1C concentrations, as shown in Figure 2 (r = 0.622, p<0.001). Using a linear regression model, the following formula was extracted:
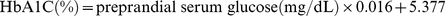



On the other hand, there was no significant association between HbA1C and log hsCRP levels (r = 0.029, p = 0.744).

### Comparisons of clinical and biochemical parameters among patients according to HbA1C levels

To explore whether patients with good and poor glycemic control had different clinical and biochemical parameters, the study subjects were divided into tertile groups according to their mean of HbA1C levels. The mean HbA1C levels in the 1^st^, 2^nd^, and 3^rd^ tertiles were 6.3% (range, 5.2–6.7), 7.1% (6.8–7.5), and 8.5% (7.6–13.3), respectively. The percentage of patients in each tertile with HbA1C levels within the levels recommended by the American Diabetes Association [2] were 100%, 42.6%, and 0% in the 1^st^, 2^nd^, and 3^rd^ tertiles, respectively. The proportion of male patients was significantly higher in the 1^st^ and 2^nd^ tertiles than in the 3^rd^ tertile (p<0.05). Serum albumin was significantly lower in the 3^rd^ tertile than the 1^st^ tertile (p<0.05). In contrast, there were no significant differences among the three tertiles in age, proportion of diabetes as the cause of ESRD, CCI score, BMI, systolic and diastolic blood pressure, hemoglobin, creatinine, calcium, phosphorus, total cholesterol, log-transformed iPTH, and log hsCRP levels. Residual renal function, Kt/V_urea_, and nPCR were also comparable among the three groups. In addition, there was no difference in the frequencies of hypoglycemic events among tertiles (Table 1).

### Causes of death among patients according to HbA1C levels

The causes of death for each tertile are shown in Table 2. Overall, cardiovascular disease and infection were the most common causes of death (18.5 per 1000-patient-year for each). However, while deaths from cardiovascular diseases occurred at similar frequencies across tertiles, deaths from infection increased according to increasing HbA1C tertiles. Therefore, compare to the 1^st^ tertile, all-cause mortality increased in the 2^nd^ tertile and even more increased in the 3^rd^ tertile. While cardiovascular disease was the most common cause of death in the 1^st^ (12.2 per 1000-patient year) and 2^nd^ (22.0 per 1000-patient-year) tertiles, infection was the leading cause of death in the 3^rd^ tertile (42.6 per 1000-patient-year).

**Table 2:** Differences in the cause of death among tertiles.

Cause of death	I	II	III	Total
Cardiovascular disease	12.2	22.0	21.3	18.5
Infection	0	16.5	42.6	18.5
Other (Malignancy, Bleeding)	6.1	11.0	7.1	8.2
All-cause	18.3	49.5	71.0	45.2

per 1000-patient-year.

### Factors influencing all-cause mortality

In univariate Cox proportional hazards analysis, age [hazard ratio (HR), 1.07 per 1 year; 95% confidence interval (CI), 1.02–1.13; p = 0.01], CCI score (HR, 1.82 per 1 point; 95% CI, 1.24–2.67; p<0.01), and log hsCRP (HR, 1.43 per 1 unit; 95% CI, 1.10–1.87; p<0.01) were significantly associated with all-cause mortality in diabetic PD patients, whereas there were significant inverse correlations between all-cause mortality and variables such as MAP (HR, 0.95 per 1 mmHg; 95% CI, 0.92–0.99; p = 0.013) and serum creatinine [HR, 0.83 per 1 mg/dL; 95% CI, 0.68–0.99; p = 0.045].

### Impact of HbA1C levels on all-cause mortality

**Figure 3:**
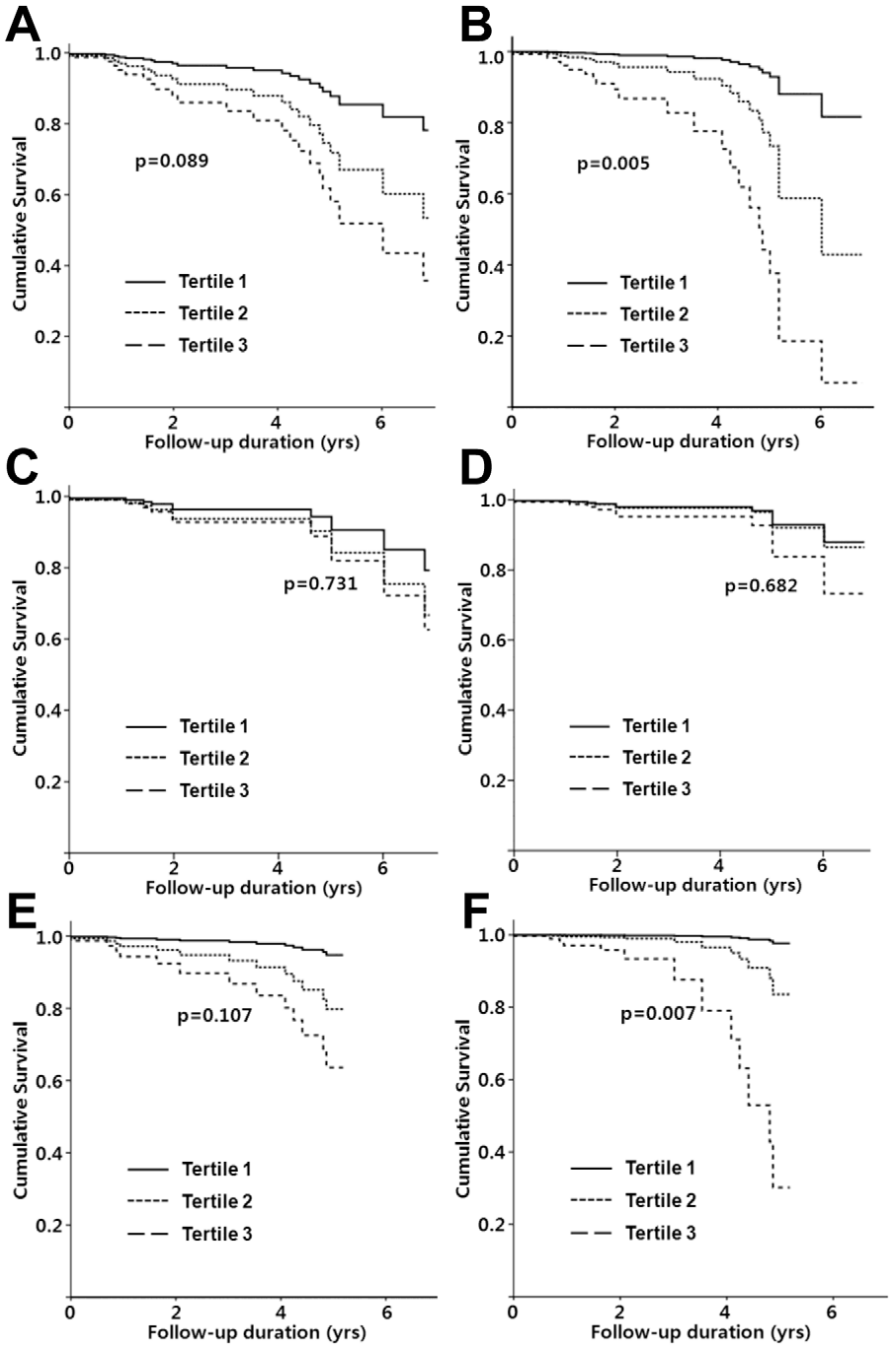
Comparison of cumulative survival among tertiles, plotted by Cox proportional hazards analysis. (A–B) Comparison of all-cause mortality among tertiles in unadjusted (A) and fully-adjusted model (B). (C–D) Comparison of cardiovascular mortality among tertiles in unadjusted (C) and fully-adjusted model (D). (E–F) Comparison of non-cardiovascular mortality among tertiles in unadjusted (E) and fully-adjusted model (F).

Although all-cause mortality in the 3^rd^ tertile group was significantly higher than in the 1^st^ tertile (HR, 4.18; 95% CI, 1.15–15.21; p = 0.030), higher HbA1C levels were not associated with all-cause mortality in the unadjusted Cox proportional hazards analysis (p for trend = 0.089) (Table 3 and Figure 3). Using case-mix and fully-adjusted models, however, there was a significant association between the mean HbA1C levels and all-cause mortality (p for trend, 0.020 and 0.005, respectively). In the case-mix model, there were 2.22- and 6.08-fold increases in the risk of all-cause mortality in the 2^nd^ (95% CI, 0.58–8.41; p = 0.243) and the 3^rd^ tertiles (95% CI, 1.58–23.49; p = 0.009), respectively, compared to the 1^st^ tertile. The risk of all-cause mortality increased further in the 2^nd^ (HR, 4.16; 95% CI, 0.91–18.94; p = 0.065) and 3^rd^ tertiles (HR, 13.16; 95% CI, 2.67–64.92; p = 0.002) using the fully-adjusted model.

**Table 3:** Risk of all-cause, cardiovascular, and non-cardiovascular mortality among tertiles (n = 140).

	All-cause	Cardiovascular	Non-cardiovascular
Model	HR (95% CI)	HR (95% CI)	HR (95% CI)
Unadjusted	P for trends 0.089	P for trends 0.731	P for trends 0.107
Tertile I	1.00	1.00	1.00
Tertile II	2.55 (0.69–9.41)	1.74 (0.32–9.54)	4.16 (0.49–35.65)
Tertile III	4.18 (1.15–15.21)	2.02 (0.33–12.17)	8.31 (1.02–51.57)
Case-mix	P for trends 0.020	P for trends 0.532	P for trends 0.029
Tertile I	1.00	1.00	1.00
Tertile II	2.22 (0.58–8.41)	1.76 (0.30–10.21)	3.01 (0.34–26.78)
Tertile III	6.08 (1.58–23.49)	3.09 (0.43–22.28)	13.03 (1.47–85.34)
Fully-adjusted	P for trends 0.005	P for trends 0.682	P for trends 0.007
Tertile I	1.00	1.00	1.00
Tertile II	4.16 (0.91–18.94)	2.80 (0.28–28.40)	7.67 (0.68–86.37)
Tertile III	13.16 (2.67–64.92)	2.46 (0.15–39.67)	51.24 (3.85–340.35)

Case-mix model is adjusted for age, gender, year of PD start, Charlson comorbidity index score. Fully-adjusted model is adjusted for mean arterial pressure, albumin, serum creatinine, and log-transformed hsCRP, in addition to all variables which were used in case-mix model.

### Impact of HbA1C levels on cardiovascular mortality

The risk of cardiovascular mortality was comparable among the three tertiles in the unadjusted, case-mix, and fully-adjusted models (p for trend, 0.731, 0.532, and 0.682, respectively) (Table 3 and Figure 3).

### Impact of HbA1C on non-cardiovascular mortality

The risk of non-cardiovascular mortality increased in the 2^nd^ (HR, 4.16; 95% CI, 0.49–35.65; p = 0.194) and 3^rd^ tertiles (HR, 8.31; 95% CI, 1.02–51.57; p = 0.048) compared to the 1^st^ tertile, but this trend failed to reach statistical significance (p for trend, 0.107). In the case-mix model, there were 3.01- and 13.03-fold increases in the risk of non-cardiovascular mortality in the 2^nd^ (95% CI, 0.34–26.78; p = 0.323) and the 3^rd^ tertiles (95% CI, 1.47–85.34; p = 0.021), respectively, compared to the 1^st^ tertile (p for trend = 0.029). The risk of non-cardiovascular mortality significantly increased further in the 2^nd^ (HR, 7.67; 95% CI, 0.68–86.37; p = 0.099) and 3^rd^ tertiles (HR, 51.24; 95% CI, 3.85–340.35; p = 0.003) using the fully-adjusted model (p for trend = 0.007), as shown in Table 3 and Figure 3.

### Impact of HbA1C on clinical outcomes in diabetic PD patients, whose etiology of ESRD was diabetic nephropathy

To elucidate whether the impact of glycemic control on clinical outcomes was comparable in diabetic PD patients whose etiology of ESRD was diabetic nephropathy, we performed additional analysis with the data of these patients (n = 119). The risk of all-cause mortality was not significantly increased in the 2^nd^ (HR, 1.40; 95% CI, 0.35–5.60; p = 0.638) and 3^rd^ tertiles (HR, 3.69; 95% CI, 0.99–13.70; p = 0.051) compared to the 1^st^ tertile in the unadjusted model (p for trend = 0.065). In the case-mix model, however, there were 1.2- and 4.68-fold increases in the risk of all-cause mortality in the 2^nd^ (95% CI, 0.29–5.05; p = 0.328) and 3^rd^ tertiles (95% CI, 1.19–18.44; p = 0.028), respectively, compared to the 1^st^ tertile (p for trend = 0.023). The risk of all-cause mortality increased further in the 2^nd^ (HR, 3.30; 95% CI, 0.57–19.28; p = 0.185) and 3^rd^ tertiles (HR, 12.71; 95% CI, 2.23–42.39; p = 0.004) using the fully-adjusted model (p for trend = 0.010). Meanwhile, there was a significant increase in the risk of non-cardiovascular mortality in the 2^nd^ (HR, 4.62; 95% CI, 0.33–44.42; p = 0.255) and 3^rd^ tertiles (HR, 33.92; 95% CI, 2.80–120.22; p = 0.003) relative to the 1^st^ tertile using the fully-adjusted model (p for trend = 0.006), while the risk of cardiovascular mortality was comparable among the three tertiles in the unadjusted, case-mix, and fully-adjusted models (p for trend, 0.898, 0.920, and 0.498, respectively) (Table 4).

**Table 4:** Risk of all-cause, cardiovascular, and non-cardiovascular mortality among tertiles in patients whose etiology of ESRD was diabetic nephropathy (n = 119).

	All-cause	Cardiovascular	Non-cardiovascular
Model	HR (95% CI)	HR (95% CI)	HR (95% CI)
Unadjusted	P for trends 0.065	P for trends 0.898	P for trends 0.107
Tertile I	1.00	1.00	1.00
Tertile II	1.40 (0.35–5.60)	1.13 (0.19–6.80)	4.16 (0.49–35.65)
Tertile III	3.69 (0.99–13.70)	0.65 (0.06–7.57)	8.31 (1.02–67.57)
Case-mix	P for trends 0.023	P for trends 0.920	P for trends 0.013
Tertile I	1.00	1.00	1.00
Tertile II	1.21 (0.29–5.05)	1.47 (0.23–9.56)	1.43 (0.14–14.47)
Tertile III	4.68 (1.19–18.44)	1.40 (0.10–19.97)	9.22 (1.10–77.37)
Fully-adjusted	P for trends 0.010	P for trends 0.498	P for trends 0.005
Tertile I	1.00	1.00	1.00
Tertile II	3.30 (0.57–19.28)	1.29 (0.28–14.54)	4.62 (0.33–44.42)
Tertile III	12.71 (2.23–42.39)	0.60 (0.10–28.57)	33.92 (2.80–120.22)

Case-mix model is adjusted for age, gender, year of PD start, Charlson comorbidity index score. Fully-adjusted model is adjusted for mean arterial pressure, albumin, serum creatinine, and log-transformed hsCRP, in addition to all variables which were used in case-mix model.

## Discussion

In this prospective observational study on 140 incident diabetic PD patients from a single center, we found that poor glycemic control was associated with increased risk of mortality in diabetic PD patients, after adjusting for confounding factors. However, there were no differences in cardiovascular mortality rates among patients with different levels of glycemic control. These findings suggest that diabetic patients on PD could benefit from strict glycemic control, even if such control may not decrease cardiovascular mortality.

Tight glycemic control has been demonstrated to prevent the development and progression of microvascular complications and to be associated with reduced risk of coronary heart disease in diabetic patients [3]–[5]. In addition, previous studies have shown that high blood glucose concentrations are associated with increased incidence of cardiovascular diseases in patients with DM [6], [7]. Based on these findings, it has been supposed that strict glucose control could exert a beneficial impact on the survival and cardiovascular outcome in diabetic patients, drawing up current guidelines of a target HbA1C level of 7.0% or less for most DM patients. Against these expectations, however, several recent studies showed that there was no beneficial effect of tight glycemic control on the cardiovascular morbidity and mortality in type 2 DM patients without advanced renal failure [8]–[10].

Findings regarding the impact of glycemic control on the outcomes of DM patients on dialysis have also been inconsistent. An analysis of 23,618 American diabetic HD patients showed that the adjusted risk for all-cause mortality in patients with HbA1C ≥10.0% was 1.41-higher than patients with HbA1C in the 5–6% range [13]. Most previous studies including East Asian diabetic patients on HD also found that poor glycemic control was associated with reduced surivival, which agrees with the results of our study [11], [21]. In contrast, a recent study by Shurraw et al [14] showed that higher blood glucose and HbA1C levels were not associated with mortality in 1,484 incident HD patients in Canada. These conflicitng results may be attributed to the differences in ethnicity, body size, the duration of dialysis, and the definition of good glycemic control.

Meanwhile, there has been only one study conducted among PD patients, and it has revealed that poor glycemic control was associated with poor survival in diabetic PD patients [15]. However, few Asian patients were included in that study, and the impact of glycemic control on patient outcomes among Asian diabetic PD patients is still unclear. Although another report by Wu et al [16], which was conducted among Asian PD patients, revealed that glycemic control before starting dialysis was a predictor of survival for type 2 diabetic patients on PD, the importance of glycemic control after starting dialysis was not evaluated. Since PD fluid contains extremely high concentrations of glucose, we hypothesized that the glycemic control in PD patients would be different from the predialysis state. Therefore, we determined glycemic control by using average HbA1C levels during the 1^st^ year after beginning PD, which were supposed to better reflect overall serum glucose concentrations. To exclude the possibility of improper hyperglycemia, moreover, the HbA1C levels around the time of acute illness or when taking medications that could affect serum glucose concentrations were omitted from the mean HbA1C levels.

In this study, poor glycemic control was associated with deleterious outcomes but not cardiovascular mortality which is the most common cause of death in ESRD patients undergoing dialysis. Consistent with these results, most previous studies have failed to demonstrate that good glycemic control improves cardiovascular survival in patients with long duration of DM [8]–[10]. Since most diabetic ESRD patients already have advanced microvascular and macrovascular complications, there might be a “point of no return”, after which patient outcomes are not affected by strict glycemic control. Is it also relevant for diabetic ESRD patients whose primary renal diagnosis is not diabetic nephropathy? To answer this issue, we performed an additional subgroup analysis in patients whose primary renal disease was diabetic nephropathy. In result, the all-cause and non-cardiovascular mortality was also significantly higher in the 3^rd^ tertile group compared to the 1^st^ tertile group, whereas the risk of cardiovascular mortality was not different among groups, which were similar to the results with all diabetic PD patients. Therefore, it is surmised that “point of no return” theory can be applied at least to PD patients in whom the etiology of ESRD was diabetic nephropathy. Meanwhile, a previous American report [13] observed a significantly higher cardiovascular mortality in patients with HbA1C ≥10.0%, while the rates were comparable among patients with HbA1C levels between 5.0% and 10.0%, suggesting that only extremely uncontrolled hyperglycemia may affect cardiovascular outcomes. Only 4 patients (2.8%) in our study sample had mean HbA1C levels greater than 10.0%, and therefore this effect might not be reflected in our study. There is also another possibility that “survival bias” could be involved in the results of cardiovascular mortality. In our study subjects, CVD was less in tertile II (21.3%), as compared with tertile I (39.1%) and tertile III (34.0%). One explanation to this observation is that patients with moderate glycemic control died of cardiovascular events even before starting PD and reaching at poorer glycemic states, and those who have reached to the 3^rd^ tertile survived from any cardiovascular events.

This study revealed that patients with poor glycemic control had significantly higher non-cardiovascular mortality, mainly due to infection. Similarly, a Taiwanese study [16] and another Korean study on diabetic PD patients [22] also found that the proportion of mortality from infection was high and comparable to that from cardiovascular diseases in their subjects, which raises several questions. Why is there a difference in the proportion of mortality from infection between diabetic HD and PD patients? Why infection-related mortality is influenced by the degree of glycemic control? While the answers are not clear, mounting evidence has shown that diabetic PD patients may be more vulnerable to infections. Frequently exchanging PD fluid could eliminate or dilute phagocytes and immunoglobulins normally present in the peritoneal cavity. In fact, the amount of removed immunoglobulin G and C3 through PD is reported to be significantly greater in DM than non-diabetic patients [23]. Moreover, hypertonic glucose solution used for PD could make patients susceptible to infection, especially in diabetic patients. It is well known that 60 to 80% of glucose in dialysate is systemically absorbed by diffusion and lymphatic absorption during a 6-hour dwell, which makes strict glycemic control more difficult in PD patients. These local and systemic hyperglycemic conditions have been suggested to be able to modify cytokine production and phagocytotic activity of immune cells by several mechansims, including hyperosmotic stress [24]. Furthermore, the production of advanced glycation endproducts can increase under hyperglycemic conditions, resulting in increased interaction between advanced glycation endproducts and their receptors, which can in turn increase inflammatory response [25].

Several shortcomings of this study should be discussed. First, as a single center study, it is subject to the biases inherent to this study design. In addition, 145 patients out of the total incident PD patients (n = 810) had diabetes, which corresponds to only 18% of incident PD patients. Considering the fact that 35 to 40% of incident ESRD patients in Korea from 2001 to 2009 had diabetes [26], we could not completely affirm that there was no selection bias even though it was not intentional. We surmise that the discrepancy in the proportion of DM patients between incident HD and PD patients in our institute may be partially attributed to our physician's tendency to hesitate to perform PD in DM patients, especially in whom predialysis blood glucose control was not appropriate. In fact, only 2.8% of this study subjects had mean HbA1C greater than 10.0%, which was much lower than 6.6% of enrolled patients in an American report [15]. Second, besides serum glucose and HbA1C levels, laboratory values at 3 months after starting PD were used for analyses in most cases. Therefore, the changes of confounding factors during the follow-up were not reflected. Third, diabetic ESRD patients, whose cause of ESRD was not diabetic nephropathy, could have different response to poor glycemic control. However, due to a small number of these patients (n = 21), subgroup analysis was not able to be performed for this issue. Lastly, there are some limitations for using HbA1C levels as a surrogate marker of glycemic control in dialysis patients. However, tests for better surrogate markers such as glycoalbumin are not widely performed and have been available in our institute only after 2009.

In conclusion, this study demonstrated that poor glycemic control was associated with higher all-cause mortality, mainly non-cardiovascular mortality represented by infection-related deaths, in diabetic PD patients. These findings suggest that better glycemic control may improve the outcome of these patients. Clinical trials are needed to better examine the impact of strict glycemic control on survival in diabetic PD patients.
